# Population Structure and Selection Signal Analysis of Nanyang Cattle Based on Whole-Genome Sequencing Data

**DOI:** 10.3390/genes15030351

**Published:** 2024-03-11

**Authors:** Yan Zhang, Zhitong Wei, Man Zhang, Shiwei Wang, Tengyun Gao, Hetian Huang, Tianliu Zhang, Hanfang Cai, Xian Liu, Tong Fu, Dong Liang

**Affiliations:** 1 College of Animal Science and Technology, Henan Agricultural University, Zhengzhou 450046, China; zhangy20210000@163.com (Y.Z.); weizhitong1206@163.com (Z.W.); 18338210615@139.com (M.Z.); wangshiwei202402@163.com (S.W.); dairycow@163.com (T.G.); xh722109@163.com (H.H.); zhangtianliu@foxmail.com (T.Z.); caihanfang.cool@163.com (H.C.); futong2004@126.com (T.F.); 2 Henan Animal Husbandry Station, Zhengzhou 450008, China; liuxian641@163.com

**Keywords:** Nanyang cattle, whole-genome resequencing, genetic diversity, population structure

## Abstract

With a rich breeding history, Nanyang cattle (NY cattle) have undergone extensive natural and artificial selection, resulting in distinctive traits such as high fertility, excellent meat quality, and disease resistance. This makes them an ideal model for studying the mechanisms of environmental adaptability. To assess the population structure and genetic diversity of NY cattle, we performed whole-genome resequencing on 30 individuals. These data were then compared with published whole-genome resequencing data from 432 cattle globally. The results indicate that the genetic structure of NY cattle is significantly different from European commercial breeds and is more similar to North–Central Chinese breeds. Furthermore, among all breeds, NY cattle exhibit the highest genetic diversity and the lowest population inbreeding levels. A genome-wide selection signal analysis of NY cattle and European commercial breeds using Fst, θπ-ratio, and θπ methods revealed significant selection signals in genes associated with reproductive performance and immunity. Our functional annotation analysis suggests that these genes may be responsible for reproduction (*MAP2K2*, *PGR*, and *GSE1*), immune response (*NCOA2*, *HSF1*, and *PAX5*), and olfaction (*TAS1R3*). We provide a comprehensive overview of sequence variations in the NY cattle genome, revealing insights into the population structure and genetic diversity of NY cattle. Additionally, we identify candidate genes associated with important economic traits, offering valuable references for future conservation and breeding efforts of NY cattle.

## 1. Introduction

The worldwide domestication of cattle includes classifications into humpless taurine (*Bos taurus*) and humped indicine (*Bos indicus*), both falling under the Bovis genus in the Artiodactyla order [1]. China possesses abundant germplasm resources for cattle and has a rich history of breeding, prolonged hybridization events have enabled their offspring to adapt to diverse environments. Whole-genome sequencing (WGS) technology is widely used to examine the genetic structure of populations and identify important economic traits [2]. The progress in high-throughput sequencing has greatly expanded our ability to identify these selection characteristics [3]. In recent years, there has been a growing trend toward using WGS to study the adaptability of local cattle breeds. Local cattle breeds, characterized by high fertility, excellent meat quality, and disease resistance, are manifested in genomic regions. Uncovering the genomic variation characteristics of local cattle breeds can guide adjustments to the genetic breeding strategy of commercial breeds, enhancing disease resistance and economic value.

Nanyang cattle (NY cattle), one of China’s five outstanding cattle breeds, played a pivotal role in the country’s agricultural production during the farming era. Recognizing the significance of preserving and genetically enhancing local breeds, China has established cattle breeding and scientific research institutions. NY cattle hold an important position on the National Catalogue of Livestock and Poultry Genetic Resources by the Ministry of Agriculture and Rural Affairs of China [4]. The current research on the population structure of NY cattle confirms that they primarily originated from hybridization between Bos taurus and Bos indicus [5]. Recent investigations into economic traits of NY cattle have predominantly focused on the exploration of candidate genes. For instance, research has delved into the impact of genes like *SIRT1* [6] and *VEGF* [7] on body weight gain, *FBXO32* [8] and *PAX3* [9] on body length, and *MYOD1* [10] on meat quality. However, there is currently no identification of genes associated with adaptive traits in NY cattle on WGS data.

This study focused on NY cattle, analyzing their population structure and genetic diversity to assess their characteristics. Additionally, by comparing them with European commercial breeds, we identified candidate genomic regions in NY cattle that significantly differ from those in European commercial breeds. This exploration aimed to investigate candidate genes associated with the characteristics of NY cattle, providing a theoretical basis for the conservation and utilization of NY cattle genetic resources.

## 2. Materials and Methods

### 2.1. Animals

A total of 462 cattle samples were utilized, including 30 NY cattle collected in Nanyang City, China, and 432 samples (Table 1) of ordinary cattle downloaded from a public database: BGVD (http://animal.omics.pro/code/index.php/BosVar, accessed on 21 March 2023) [11].

### 2.2. Sequencing

Genomic DNA was extracted from blood samples of NY cattle, using the phenol–chloroform method. The integrity and yield of genomic DNA were assessed and verified through agarose gel electrophoresis and NanoDrop spectrophotometry, respectively, prior to whole-genome resequencing (Supplementary Table S1). The DNBSEQ-T7 platform was applied to construct paired-end reads with an insert size of 150 bp for each individual’s genomic DNA, followed by sequencing. Trimmomatic was utilized to filter the generated fastq data, producing clean reads. The clean reads were aligned to the reference genome (ARS-UCD1.3), using the BWA-MEM algorithm [12] with the default parameters. PCR duplicates were removed using the MarkDuplicates module in the Picard toolkit (https://broadinstitute.github.io/picard/index.html, accessed on 27 March 2023). Base quality recalibration was performed using the BaseRecalibrator module of GATK v 3.8 [13]. The UnifiedGenotyper module of GATK was then applied for variant detection, with post-detection filtering parameters set as follows: ‘QD < 2.0, FS > 60.0, MQ < 40.0, MQRankSum < −12.5, ReadPosRankSum < −8.0, AF < 0.01, DP < 800’. We obtained a total of 30,738,668 SNPs. We converted the BGVD sequencing data to the reference genome ARS-UCD1.3, using the liftOver v398 software, and merged the two VCF files, using the merge option in bcftools v 1.5 software. After data merging, we obtained a total of 69,393,991 SNPs. The variant data file was converted from VCF to PLINK format, using VCFtools v 0.1.16 [14]. During quality control filtering, SNPs with minor allele frequencies < 0.05 and departure from Hardy–Weinberg equilibrium <10^−6^ were excluded, along with individuals with missing genotypes >10%. After quality control, a total of 23,872,695 high-quality SNPs were retained.

### 2.3. Phylogenetic and Population Structure Analysis

To investigate the population structure of NY cattle, we merged data from 30 NY cattle with publicly available data from 432 cattle. Using these merged SNP data, we conducted a population structure analysis. We utilized PLINK v1.90 [15] to compute the IBS distance matrix between populations. The NJ (neighbor-joining) method was employed to construct the phylogenetic tree, and the resulting tree file in nwk format was generated using the ATCG (http://www.atgc-montpellier.fr/fastme/, accessed on 8 October 2023) website. Subsequently, we visualized the tree using FigTree v1.4.4 (http://tree.bio.ed.ac.uk/software/figtree/, accessed on 12 October 2023). After removing SNP sites with the high levels of pair-wise linkage disequilibrium (LD) using PLINK v1.90, we conducted a principal component analysis (PCA), utilizing its pca option. Following this, we visualized the outcomes using the ggplot2 R package v3.4.2 (https://ggplot2.tidyverse.org/, accessed on 12 September 2023). A population structure analysis was conducted using ADMIXTURE v1.3.0 [16], with K representing the number of ancestral populations; the range for K was set from 2 to 4.

### 2.4. Runs of Homozygosity, Linkage Disequilibrium, Genetic Diversity, and Inbreeding Coefficient Detection

The detection of Runs of Homozygosity (ROHs) was performed using the --homozyg option implemented in PLINK v1.90. The sliding window method was applied to identify ROH on autosomes, and the ROH segments were categorized into 5 size ranges, namely 0~0.5 Mb, 0.5~1 Mb, 1~2 Mb, 2~4 Mb, and >4 Mb, for subsequent analysis [17]. The data obtained from this analysis were visualized using the ggplot2 R package v3.4.2. The inbreeding coefficient (FROH) was subsequently calculated based on the obtained ROH results. The calculation formula for the FROH based on ROH estimation is $FROH=\frac{\sum i\left(LROHi\right)}{Lauto}$. Here, LROHi represents the length of ROH for individual i, and Lauto denotes the coverage length of autosomal SNPs. PopLDdecay v3.42 [18] was applied to conduct an LD analysis on the genotype data of each breed separately. By calculating the LD value (r2) between two SNPs and their physical distance, LD decay curves were plotted, and the LD decay rates were compared among different breeds. In the computation of nucleotide diversity, we applied VCFtools to estimate values within a window size of 50 kb and a step size of 20 kb.

### 2.5. Selective Sweep Identification

We applied PLINK v1.90 for quality control of the data files in VCF format and conducted a genome-wide scan of the NY cattle. Three different statistical methods were applied, namely the Fst, θπ-ratio, and θπ. The Fst and θπ-ratio were used to measure the degree of population differentiation between NY and the Europe commercial breeds (Angus, Hereford, Charolais, Jersey, and Simmental), while θπ was applied to detect selective traits within NY cattle. To calculate Fst values, we utilized VCFtools with a sliding-window approach, setting 50 kb windows with a 25 kb step. The θπ was estimated based on a sliding-window approach, with windows of 50 kb and a step of 25 kb, using VCFtools. To obtain the θπ-ratio, we applied the formula θπ (NY)/θπ (Europe commercial breeds). The windows in the top 1% were considered candidate selective regions and subjected to gene annotation. Through gene annotation of these candidate regions, we identified candidate genes. Online KEGG pathway enrichment analysis was conducted using KOBAS (http://bioinfo.org/kobas/genelist/, accessed on 1 November 2023) to determine their significant enrichment in functional categories and signaling pathways. Furthermore, we incorporated the genes annotated from the top 1% windows detected by the θπ analysis to identify variations within the NY cattle. We then conducted a joint analysis using the candidate genes obtained from the three methods. This analysis aimed to validate the expression levels of these candidate genes and investigate their potential roles.

## 3. Results

### 3.1. Genome Sequencing and Population Structure Analysis

**Figure 1:**
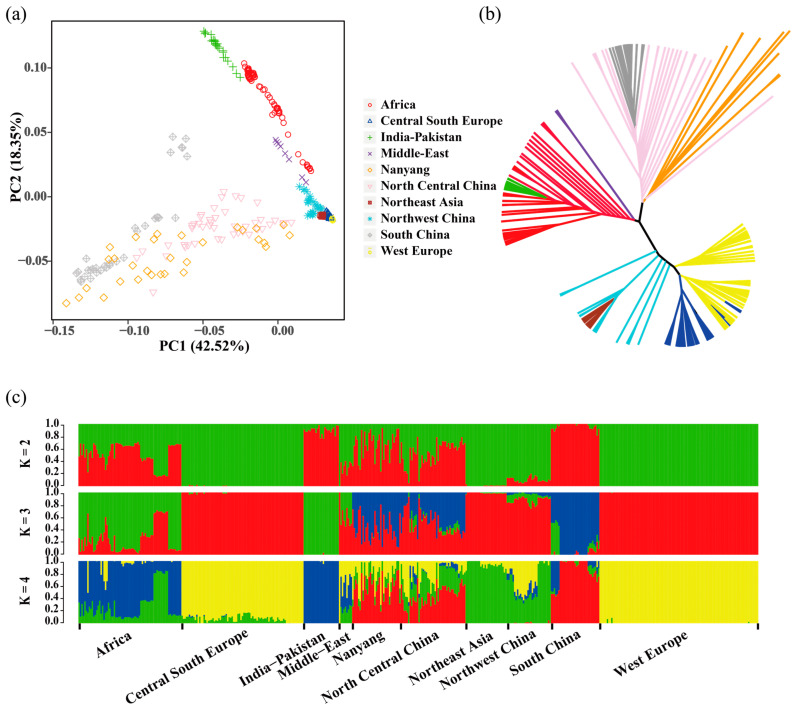
Genetic structure analysis of the Nanyang population. (**a**) Principal component analysis based on whole-genome sequencing data. (**b**) Phylogenetic analysis constructed based on genetic distance. (**c**) Model-based clustering of breeds was performed using ADMIXTURE, with K values ranging from 2 to 4.

To explore the population structure of NY cattle, we conducted a population structure analysis using these SNP data. The results indicate that the NY cattle primarily clusters with the North–Central China and South China breeds (Figure 1a,b), aligning with the geographical distribution of populations. In the ADMIXTURE analysis (Figure 1c), it can be observed that NY cattle exhibit a complex genetic background. When K = 2, NY cattle are most similar to African and North–Central China breeds and can be distinctly differentiated from European breeds; when K = 3 and K = 4, NY cattle are most similar to North–Central China breeds and are clearly distinct from African breeds.

### 3.2. Patterns of Genomic Variation

**Figure 2:**
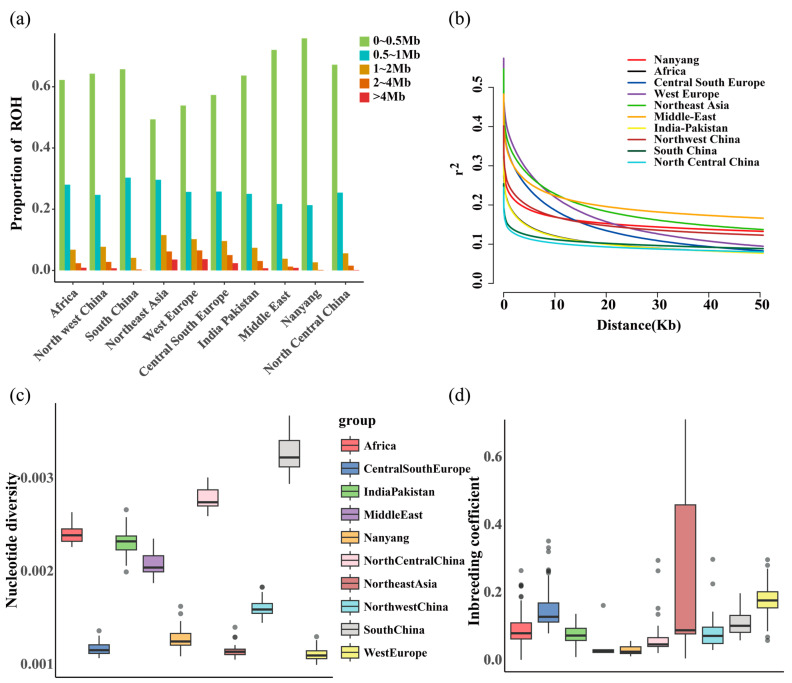
Analysis of ROH distribution patterns and genetic diversity. (**a**) Proportion of ROH segments across the entire genome in different breeds. (**b**) Linkage disequilibrium analysis based on whole-genome resequencing. (**c**) Nucleotide diversity across different breeds. The black line in the boxplot is the median line, and the outside points are outliers. (**d**) Average inbreeding coefficients for each breed. The black line in the boxplot is the median line, and the outside points are outliers.

To investigate the distribution patterns of Runs of Homozygosity (ROHs) and nucleotide diversity levels in NY cattle relative to other breeds, we classified ROH segments from all breeds into five levels (0~0.5 Mb, 0.5~1 Mb, 1~2 Mb, 2~4 Mb, and >4 Mb). The results of the ROH analysis (Figure 2a) reveal that, in the NY cattle and other Chinese breeds, the proportion of short ROH segments is generally higher than that in European commercial breeds. Specifically, in the NY cattle, the proportion of ROH segments with lengths between 0 and 0.5 Mb is at a relatively high level compared to all breeds, while the proportion of ROH segments >4 Mb is the lowest among all breeds, lower than in European commercial breeds. The results of linkage disequilibrium analysis (Figure 2b) show that breeds in China, including NY cattle, generally exhibit a faster LD decay rate, whereas European commercial breeds tend to have a slower LD decay rate. In the nucleotide diversity analysis results (Figure 2c), the nucleotide diversity levels in European commercial breeds and Hanwoo breeds are notably lower than those in NY cattle and other Chinese breeds, indicating lower genetic diversity levels. NY cattle exhibit the highest nucleotide diversity level among all breeds. Our analysis of population inbreeding (Figure 2d) reveals that European commercial breeds and Hanwoo breeds generally have higher levels of inbreeding compared to NY cattle and other Chinese breeds, with NY cattle showing the lowest levels of population inbreeding.

### 3.3. Genome-Wide Selective Sweep Test and Enrichment Analysis of Genes

**Figure 3:**
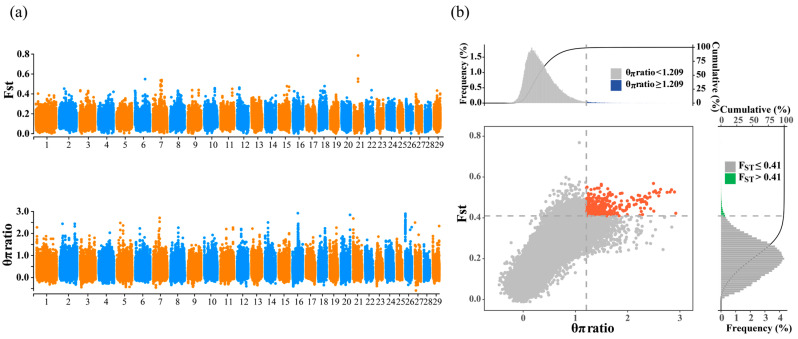
Analysis of selection signals and function enrichment in NY cattle. (**a**) Manhattan plots based on the Fst and θπ-ratio. (**b**) Selective sweep results for Fst and θπ-ratio. Fst and θπ-ratio are plotted on the *X* and *Y* axes, respectively, with horizontal and vertical gray dashed lines denoting the top 1% values of Fst (0.41) and θπ-ratio (1.209). Red dots represent significantly similar candidate selection windows identified by both methods.

To investigate the genetic differences between NY cattle and European commercial breeds, we applied the fixation index (Fst) and nucleotide diversity ratios (θπ-ratio) for the analysis (Figure 3a) between NY and the commercial breeds (Angus, Hereford, Charolais, Jersey, and Simmental). The aim was to detect genomic regions associated with selection in both NY cattle and European breeds. Candidate selective regions were identified based on outlier signals (top 1% signals) in the results of the Fst analysis and θπ-ratio analysis (Figure 3b). The Fst analysis identified a total of 682 candidate genes, while the θπ-ratio analysis identified 844 candidate genes (Supplementary Table S2). Among the significantly shared regions between the two methods, 278 candidate genes were identified. We conducted a KEGG enrichment analysis for the annotated 278 candidate genes. The results revealed two significantly enriched pathways (corrected *p*-value < 0.05): the estrogen signaling pathway and ubiquitin-mediated proteolysis. The Estrogen signaling pathway includes seven genes (*EGFR*, *NCOA2*, *MAP2K2*, *PGR*, *GABBR2*, *TGFA*, and *NCOA3*). *NCOA2* [19], *MAP2K2* [20], *PGR* [21], and *GABBR2* [22] are associated with reproduction, while *TGFA* [23] and *NCOA3* [24] are linked to immunity. The ubiquitin-mediated proteolysis pathway comprises seven genes (*UBE4B*, *FBXW11*, *ITCH*, *UBE2D1*, *FBXO2*, *PIAS4*, and *TRIP12*); *TRIP12* [25] is related to embryonic development, and *FBXW11* [26], *UBE2D1* [27], *UBE4B* [28], *FBXO2* [29], and *PIAS4* [30] are associated with immunity.

To assess genetic variation within the NY cattle population, we selected significant windows from the nucleotide diversity (θπ) analysis results as candidate selective regions, identifying a total of 657 candidate genes (Supplementary Table S2). The three methods collectively identified 20 shared candidate genes, including *NCOA2* [19], *HSF1* [31], and *PAX5* [32] associated with immunity; and *TAS1R3* [33] involved in olfactory transduction. Furthermore, the presence of genes like *MAP2K2* [20] and *GSE1* associated with reproduction underscores the multifaceted genetic adaptations within the NY cattle population. This comprehensive analysis sheds light on the intricate genetic architecture of this unique breed.

## 4. Discussion

Studying the population structure and genetic diversity of NY cattle is helpful for evaluating their genetic resources and playing an important role in protecting and utilization the NY cattle population. NY cattle is a local breed in central China, and there have been few reports on the genetic structure and genome diversity of NY cattle based on WGS data. We investigated the genetic structure and genomic diversity of NY cattle using WGS data. Combining data from cattle breeds worldwide, we found that NY cattle primarily cluster with the North–Central China and South China populations. This association may be attributed to their shared distribution in plain regions with similar topography and climatic environments. According to our ADMIXTURE analysis results, NY cattle exhibit a complex genetic background, indicating that during their extended period of husbandry, they underwent relatively frequent hybridization and improvement through the introduction of foreign cattle breeds.

We revealed the distribution patterns of ROH and genetic diversity in NY cattle, as well as various cattle breeds worldwide. ROH refers to continuous homozygous base pairs in the DNA sequence of a diploid organism, where long ROHs indicate recent inbreeding, and short ROHs suggest the influence of distant ancestors [34,35]. When comparing NY cattle to cattle breeds from around the world, including various Chinese breeds, we observed that short Runs of Homozygosity (ROHs) segments are generally more prevalent in Chinese local breeds, including NY cattle, than in European commercial breeds. Additionally, the average inbreeding coefficient of NY cattle is lower than that of European commercial breeds. These findings reflect that NY cattle, along with other locally bred Chinese cattle, exhibit a lower degree of artificial selection compared to European commercial breeds that have undergone prolonged selective breeding. The linkage disequilibrium analysis reflects the degree of linkage between genes within a species. A slower decay in LD indicates a higher level of genomic linkage, suggesting a stronger selection pressure [18]. At the whole-genome level, NY cattle exhibit a faster decay rate of LD compared to European commercial breeds. Additionally, the nucleotide diversity is significantly higher in NY cattle than in European commercial breeds. These observations reflect that NY cattle experience lower selection pressure, demonstrating a richer genetic diversity. Xia et al.’s [36] study also supports this conclusion. Due to limited artificial selection pressure and long-term adaptation to diverse environments, NY cattle retain higher genetic diversity, presenting greater breeding potential when compared to established commercial cattle breeds.

NY cattle are primarily distributed in the central region of China and have long been raised as agricultural working animals. People have tended to selectively breed robust and healthy individuals, cultivating a population with strong immune characteristics. In our study of the NY cattle population, we identified genes associated with immunity (*NCOA2*, *HSF1*, and *PAX5*). *NCOA2*, a member of the nuclear receptor coactivator family, was found by Zhong et al. [37] to stimulate T-cell activation and enhance mitochondrial function by upregulating the expression of *PGC-1α*, thereby promoting T-cell-mediated immune response. Additionally, Zhang et al. [31] demonstrated in a mouse study that the overexpression of *HSF1* reduces apoptosis of islet β cells by enhancing the expression of SIRPα. *PAX5* serves as a crucial regulator in B-cell development. A study by Lesly et al. [32] demonstrated that *PAX5* promotes PI3K signaling, contributing to the differentiation and survival of various mature B-cell types that collaborate to provide humoral immunity. These immune-related genes are likely associated with the robust immunity observed in NY cattle.

In the comparative analysis between NY cattle and European commercial cattle, we identified genes associated with fertility (*MAP2K2*, *PGR*, and *GSE1*) in NY cattle. *MAP2K2* encodes a bi-specific protein kinase within the MAP kinase family. Valerie et al. [20] reported that *MAP2K2*, along with *MAP2K1*, plays a role in the synchronous normal development of trophoblasts during placenta formation. Loss of *MAP2K2* function can impact the normal development of the placenta, leading to embryo demise during pregnancy due to placental defects. The progesterone receptor (*PGR*) is a nuclear receptor. Fang et al. [21] demonstrated that *PGR* knockout results in low fertility in tilapia, suggesting that *PGR* may play a crucial role in spermatogenesis, sperm maturation, and spermiogenesis through the activation of nuclear receptors. *GSE1* is a component of the CoREST complex. In a study involving mice [38], the loss of maternal *GSE1* led to placental dysfunction, significantly impacting mouse embryonic development. These genes are likely associated with the robust reproductive performance observed in NY cattle.

NY cattle, as valuable genetic resources in China, possess unique advantages in adaptability and genetic diversity. Against the backdrop of depleting genetic resources in the existing cattle population, research on NY cattle, a precious local breed, can aid in a comprehensive understanding of its genetic characteristics and adaptive mechanisms. The study results contribute to enhancing and utilizing the excellent breed resources of NY cattle, providing valuable insights for future research on the essential economic traits of NY cattle.

## 5. Conclusions

NY cattle, one of the excellent cattle breeds in China, are mainly distributed in the North–Central China region, characterized by their large size, strong immunity, high reproductive capacity, and gentle temperament. In this study, we explored the population structure and genomic diversity of NY cattle using SNP data from NY cattle and 432 other publicly available cattle. The population structure of NY cattle is more similar to those of cattle from South China and North–Central China regions. Compared to European commercial breeds, NY cattle exhibit higher genetic diversity. Additionally, we identified a series of candidate genes potentially associated with the reproductive performance and immune response of NY cattle. This study contributes to our understanding of the characteristics of NY cattle as a local breed and will facilitate future conservation and breeding efforts for NY cattle, providing a reference for further research on other local breed resources.

## Figures and Tables

**Table 1:** Statistical information of 432 cattle, including distribution regions, breeds, and sample sizes.

Region	Breed	Sample Size
Africa	NDama	10
Africa	Kenana	9
Africa	Ogaden	9
Africa	Boran	10
Africa	Shorthorn Zebu	10
Africa	Nganda	1
Africa	Nsongora	1
Africa	Ankole	20
Northwest China	Kazakh	9
Northwest China	Mongolian	7
Northwest China	Chaidamu	5
Northwest China	Tibetan	9
South China	Dianzhong	6
South China	Leiqiong	3
South China	Wannan	5
South China	Wenshan	8
South China	Guangfeng	4
South China	Jian	4
South China	Jinjiang	3
Northeast Asia	Yanbian	1
Northeast Asia	Hanwoo	18
Northeast Asia	Mishima	8
Northeast Asia	Kuchinoshima	1
West Europe	Angus	25
West Europe	Devon	1
West Europe	Hereford	21
West Europe	Holstein	45
West Europe	Red Angus	16
Central–South Europe	Charolais	14
Central–South Europe	Gelbvieh	21
Central–South Europe	Jersey	12
Central–South Europe	Limousin	1
Central–South Europe	Maine Anjou	6
Central–South Europe	Piedmontese	5
Central–South Europe	Salers	1
Central–South Europe	Simmental	23
India–Pakistan	Brahman	9
India–Pakistan	Gir	3
India–Pakistan	Hariana	1
India–Pakistan	Nelore	4
India–Pakistan	Sahiwal	1
India–Pakistan	Srilanka	5
India–Pakistan	Tharparkar	1
Middle East	Rashoki	9
North–Central China	Bashan	5
North–Central China	Bohai Black	5
North–Central China	Dabieshan	2
North–Central China	Jiaxian Red	5
North–Central China	Lingnan	8
North–Central China	Luxi	5
North–Central China	Nanyang	5
North–Central China	Wandong	2
North–Central China	Weining	5
North–Central China	Zaobei	5

## Data Availability

The data presented in this study are available upon request from the corresponding author.

## Supplementary Materials

The following supporting information can be downloaded at https://www.mdpi.com/article/10.3390/genes15030351/s1, Table S1: Overview of whole genome sequencing data of Nanyang cattle; Table S2: Significantly identified candidate genes by three statistical methods.
